# Mondor’s disease of the breast: a case series

**DOI:** 10.1186/s13256-021-02708-6

**Published:** 2021-04-02

**Authors:** K. Ben Hamida, M. Ghalleb, A. Triki, I. Jebir, R. Makhlouf, H. Touinsi

**Affiliations:** 1 Surgery Department, Mohamed Taher El Maamouri Hospital, Nabeul, Tunisia; 2 Faculty of Medicine of Tunis, Tunis, Tunisia

**Keywords:** Mondor’s disease, Breast disease, Superficial thrombophlebitis

## Abstract

**Background:**

Mondor’s disease of the breast (MDB) is a rare and benign disorder of the breast. It is characterized by thrombophlebitis of the superficial veins of the chest wall. Clinically, it manifests as a cord-like induration of the breast area. MDB resolves spontaneously without sequela.

**Case presentation:**

We report cases of three Caucasian African patients aged 29, 40 and 34, respectively. One patient was under progestative contraception. All the patients had a cord-like induration on the chest wall. Ultrasonography was performed in all patients and was normal in two cases and showed a thrombotic vein in one case. All the patients had symptomatic treatment with total resolution of symptoms within 1 to 4 weeks. No relapse was observed.

**Conclusion:**

MDB is benign in most cases. However, it is not to be taken lightly, because it can be the manifestation of an underlying disease such as breast cancer. The diagnosis is based on clinical findings; ultrasonography can be helpful for the diagnosis. Treatment is based on analgesic and anti-inflammatory drugs.

## Background

Mondor’s disease of the breast (MDB) is a rare and benign condition. It is characterized by thrombophlebitis of the subcutaneous venous network of the anterior chest wall. It was well described by the French surgeon Henri Mondor, who in 1939 published a case series where he described the disorder in detail [1]. In 50–60% of cases, no cause is found (primary disease), while in 40–50% of cases, some enhancing factors may be present [2].

The rarity of the disease and the absence of clear diagnostic criteria cause it to be underdiagnosed. In some cases, the presence of MDB led to the diagnosis of an underlying breast cancer. Thus, it raises the question of a possible association between breast cancer and MDB.

We aim through this study to report our experience about MDB and to review the existing literature.

## Case presentation

### Case 1

**Fig. 1:**
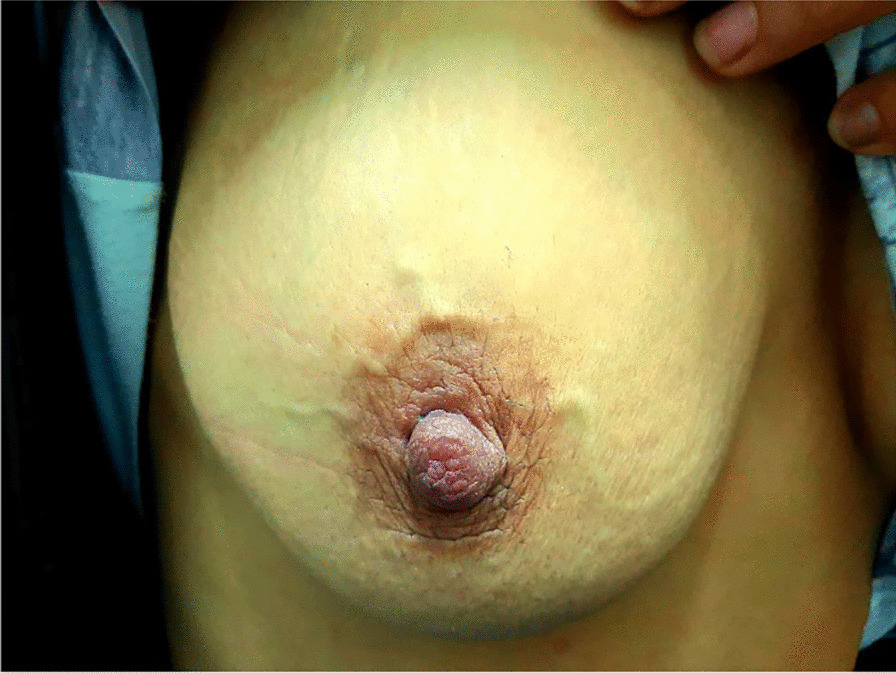
Cord-like lesion of the *circulus venosus* of Haller of the right breast

A 29-year-old Caucasian African women, with no particular past medical history, presented with 24-hour right breast pain. The patient's history revealed a local trauma 7 days before presentation. The physical exam revealed a 5-cm painless cord-like subcutaneous induration in the periareolar region of the right breast, arising in the *circulus venosus* of Haller (Fig. 1). No other signs were found.

A Doppler ultrasound was performed and was normal. The patient underwent 7 days of nonsteroidal anti-inflammatory treatment. The pain resolved within 1 week and the induration within 3 weeks. No relapse was reported after 6 months of follow-up. Screening mammogram as prescribed in the national program was performed 1 month after resolution of the symptoms and was normal.

### Case 2

**Fig. 2:**
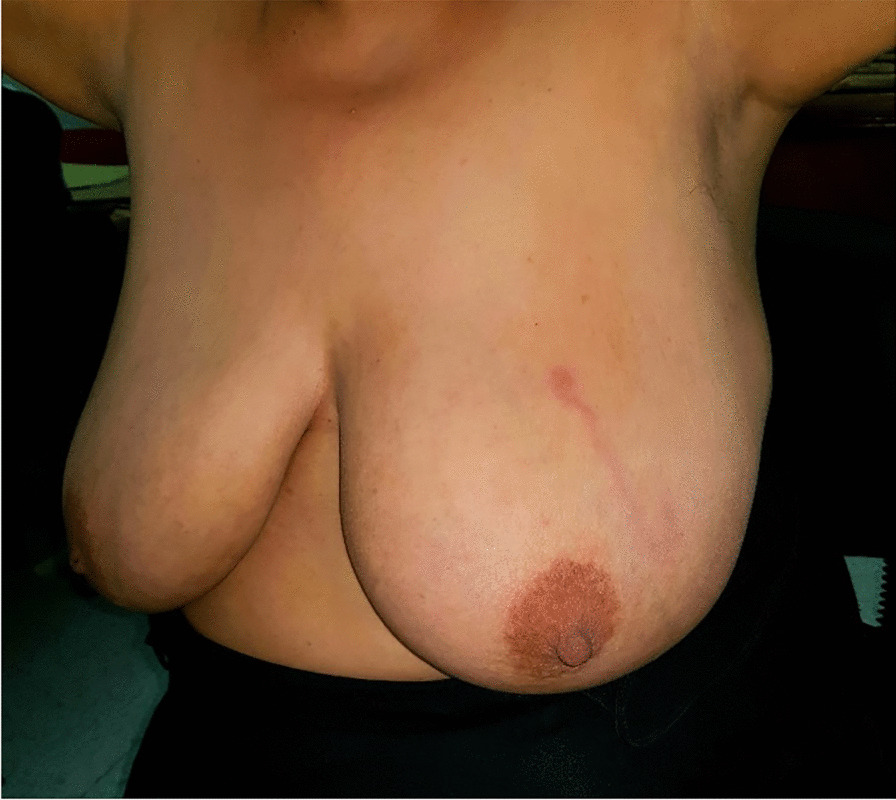
Cord-like induration of outer upper quadrant of the left breast

A 40-year-old Caucasian African women with no particular past medical history presented with a 7-day subcutaneous cord-like lesion in the left breast. The physical exam revealed a 6-cm cord-like lesion in the outer upper quadrant of the left breast (Fig. 2). There was erythema around the lesion, and the palpation was painless.

Ultrasonography showed an incompressible subcutaneous vein. The patient underwent nonsteroidal anti-inflammatory treatment, and the clinical findings disappeared after 1 month. Screening mammogram as prescribed in the national program was performed 1 month after the resolution of the symptoms and was normal.

### Case 3

**Fig. 3:**
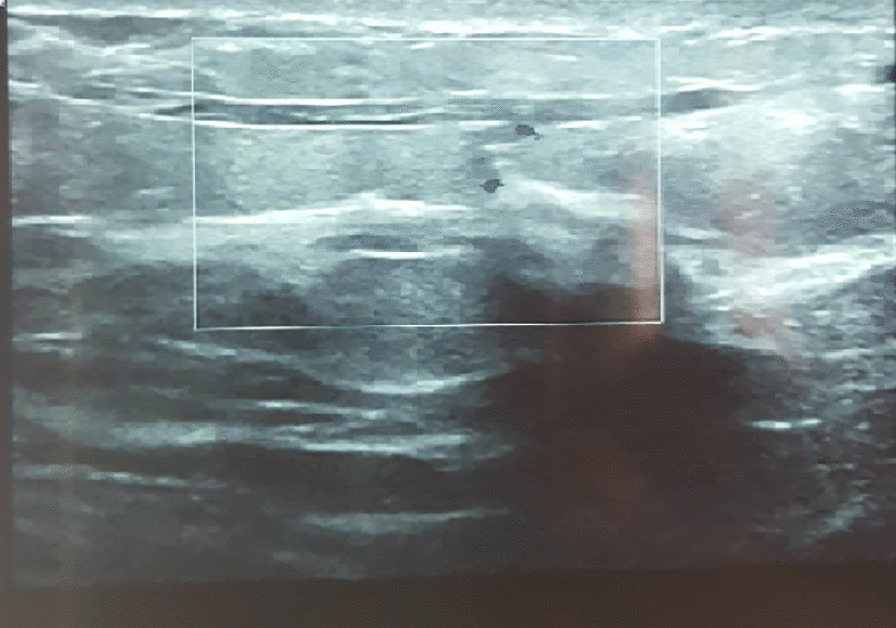
Intraluminal thrombus on sonography

A 34-year-old Caucasian African women presented with an aching right breast. She had no past medical history of breast injury or breast disease. She had been on oral estro-progestative contraception for 11 years. A 7-cm indurated and painful cord-like structure was present in the outer upper quadrant of the right breast on physical examination. The cords radiated from the retro-areolar region towards the axillary fossa. The tract adhered to the skin, with mild cutaneous retraction without erythema. On sonography, there was an intraluminal thrombus in a tubular structure (Fig. 3). No flow was present in the structure on color or spectral Doppler studies.

The patient received painkillers and a nonsteroidal anti-inflammatory drug. Complete recovery within 10 days marked the evolution of the disease. Screening mammogram as prescribed in the national program was performed 1 month after the resolution of the symptoms and was normal.

## Discussion

The incidence of MDB is unknown due to its rarity and the lack of awareness of clinical findings, especially in the primary care setting. Quéhé *et al*. found only 400 cases reported in the literature [3]. The reported sex ratio is 3:1 [4, 5]. However, it seems that female patients are more involved than male patients [2]. The average age is from 30 to 60 years, with extremes ranging from 19 to 70 years [3].

MDB is idiopathic in 50–60% of cases. In 40–50% of cases, a favoring factor can be present [2]. Many case reports suggest the presence of a relation between local trauma (such as vigorous exercise, tight brassiere, or direct injury) and the onset of the palpable cord [6].

In a retrospective study of 652 female patients who underwent aesthetic breast surgery, Goldman and Wollina [7] found three MDB cases. Catania *et al*. found eight MDB cases associated with breast cancer among a total of 68 MD cases [8].

There are few references concerning MDB during pregnancy [9, 10]. The clinical presentation is not specific, and it is unlikely that the physiological hypercoagulability is responsible for the generation of MDB. Penile lesion may be related to neoplasms, excessive sexual activity, trauma, or abstinence [11]. MD of the axilla, known as axillary web syndrome, has been documented following axillary surgery [12].

The only clinical sign of MDB is an acute, usually painful, cord-like induration arising in the chest wall area, which is the patient's main complaint. The diagnosis is based on medical history and physical examination. Further examination is usually not necessary.

Depending on the problematic vein, the induration topography may change. The lesion most commonly involves the thoracoepigastric vein and more rarely the lateral thoracic vein or superior epigastric vein [3]. Ultrasound may be needed when the clinical presentation is not suggestive. It shows incompressible tubular structures with anechoic or hypoechoic content [13]. Color Doppler can also be applied in the absence of a flow signal inside the vein [14].

Mammography is performed to determine the etiology rather than to confirm the diagnosis. In fact, since the association between MDB and breast cancer has been estimated at around 4% (1.8% in Herman's series and 12.7% in Catania’s), mammography should be considered in both genders. MDB associated with breast cancer in male patients has been reported [8, 15]. Magnetic resonance imaging and other imaging techniques are not recommended for evaluating MDB [16] and may cause unnecessary health expenses.

In general, MDB is a spontaneously regressive disease within 4 to 8 weeks, and no particular treatment is needed [17]. Warm compresses, painkillers and nonsteroidal anti-inflammatory drugs and abstinence from irritating clothing or activities constitute first-line treatment [18]. Most lesions will resolve with no other further treatment needed. However, in some cases, the cord-like induration may be palpable for several months [9].

Some authors have reported efficiency of anticoagulation treatment in the acute stage of the disease [19], such as low-molecular-weight heparin or aspirin. Local application of anticoagulation has been described in some cases of penile Mondor’s disease [20]. Local injection of triamcinolone has been successfully tested in shortening the disease in Mondor's cord after breast augmentation [21]. Surgery can be considered in the case of unbearable pain [22].

MDB is a self-limited condition that resolves spontaneously with no sequela. However, patients should be educated on the importance of follow-up.

## Conclusion

MD is a rare clinical condition for which the etiopathogenesis has yet to be elucidated. It affects more women than men. Symptoms can occur in sensitive areas, and it can become a source of discomfort for either female or male patients. Greater awareness of patients' complaints and clinical findings is required, since it is a rare disease. Usually, no further investigation is needed. Mammography is considered in women with MDB when a risk of an underlying disease is identified. Symptoms usually resolve spontaneously.

## Data Availability

All data were taken from the patient's medical folder available at the archive of our institution.
